# Networks underpinning emotion: A systematic review and synthesis of functional and effective connectivity

**DOI:** 10.1016/j.neuroimage.2021.118486

**Published:** 2021-11

**Authors:** Raphael Underwood, Eva Tolmeijer, Johannes Wibroe, Emmanuelle Peters, Liam Mason

**Affiliations:** a Psychology & Neuroscience, Department of Psychology, King's College London, Institute of Psychiatry, United Kingdom; b Max Planck Centre for Computational Psychiatry and Ageing Research, University College London, London United Kingdom; c Research Department of Clinical, Educational and Health Psychology, London, United Kingdom

**Keywords:** Functional connectivity, Emotion, Human, Healthy, Causal connectivity, Effective connectivity, Dynamic causal modeling

## Abstract

Existing models of emotion processing are based almost exclusively on brain activation data, yet make assumptions about network connectivity. There is a need to integrate connectivity findings into these models.

We systematically reviewed all studies of functional and effective connectivity employing tasks to investigate negative emotion processing and regulation in healthy participants. Thirty-three studies met inclusion criteria. A quality assessment tool was derived from prominent neuroimaging papers. The evidence supports existing models, with primarily limbic regions for salience and identification, and frontal areas important for emotion regulation. There was mixed support for the assumption that regulatory influences on limbic and sensory areas come predominantly from prefrontal areas. Rather, studies quantifying effective connectivity reveal context-dependent dynamic modulatory relationships between occipital, subcortical, and frontal regions, arguing against purely top-down regulatory theoretical models. Our quality assessment tool found considerable variability in study design and tasks employed.

The findings support and extend those of previous syntheses focused on activation studies, and provide evidence for a more nuanced view of connectivity in networks of human emotion processing and regulation.

## Highlights

• We reviewed 33 studies of functional connectivity of emotion in healthy participants.
• Our results challenge a hierarchical model of emotion processing.
• Causal connectivity analyze identify dynamic modulatory relationships between regions.
• We derive a quality tool to make recommendations addressing variability in study design.

## 1 Introduction

Neuroimaging techniques such as functional Magnetic Resonance Imaging (fMRI) provide a valuable tool for investigating how the human brain processes emotion. Functional imaging can help eschew some of the difficulties posed by self-report methods resulting from the highly subjective nature of emotion (LeDoux, 2000). Cognitive scientists and neurobiologists have increasingly turned towards emotion research in recent decades, particularly given its role in decision-making (*e.g*. Lerner et al., 2015) and well-being (Davidson, 2004), as well as in mood, personality, and psychotic disorders (*e.g*. Etkin and Wager, 2007; Mayberg et al., 1999; Minzenberg et al., 2007; Phillips and Swartz, 2014; Underwood et al., 2015). Several neurobiological models of emotion processing have been proposed (*e.g*. Buhle et al., 2014; Ochsner et al., 2012; Phillips et al., 2003), yet these have been based almost exclusively on functional activation, and so lack a refined understanding of the connectivity between implicated regions.

These models describe networks of interacting regions that contribute to identifying the emotional significance of a stimulus, the production of affective states, and emotion regulation. The literature informing these models typically employ stimuli representing distinct categories of emotion which are then contrasted during analysis (*e.g*. happy vs neutral faces). Participants are often asked to passively view these stimuli, or rate their emotional valence, or regulate their emotional response to them. Such a stimulus is initially appraised for its emotional salience by regions including the amygdala, insula, and ventral striatum (Fusar-Poli et al., 2009; Phillips et al., 2003; Sergerie et al., 2008). Subsequent production of an affective state is generated by these regions, in conjunction with the ventromedial prefrontal cortex (PFC), and the anterior cingulate cortex (Kober et al., 2008; Ochsner et al., 2012; Phillips et al., 2003). Finally, the resulting affective state is regulated up or down by primarily frontal regions including the dorsomedial, dorsolateral, ventromedial, and ventrolateral PFC, and the anterior cingulate cortex (ACC), (Buhle et al., 2014; Ochsner et al., 2012; Phan et al., 2004). These models make slightly different claims regarding the exact role of each prefrontal area. For example, the Phillips et al. (2003) does not consider the ventromedial PFC (VMPFC) to be involved in emotion regulation, while the Ochsner et al. (2012) considers this same area to aid regulation by evaluating the value of stimuli in relation to different goals. By contrast, the Buhle et al. (2014) did not find that emotion regulation consistently recruits the VMPFC, contrary to the Ochsner et al. (2012) model.

The above models uniformly predict that prefrontal areas achieve emotion regulation in a ‘top-down’ fashion, by inhibiting subcortical regions such as the amygdala (Buhle et al., 2014; Ochsner et al., 2012; Phillips et al., 2003). However, more recent papers argue that emerging anatomical and functional evidence undermines this hierarchical view of brain organization prevalent in neuroscience research (Pessoa, 2017). Rather than cortical regions regulating subcortical ones, the paper proposes that these regions form ‘integrated functional systems’ that communicate dynamically, with context-dependent changes in the direction of driving influence between regions. Whether hierarchical or dynamic, the above models make assumptions regarding the connections between regions within the emotion processing network and their driving influences on one another, without having systematically reviewed empirical connectivity findings. In the last five years, the number of connectivity studies has more than doubled and analyze that allow inferences about the directionality of connections have become more widespread. Such a review could also help evaluate the proposed hierarchical model of emotion processing, particularly by examining findings from analyze allowing inferences about directionality, such as dynamic causal modeling (DCM). The present review addresses this by reviewing functional connectivity studies of negative emotion processing and emotion regulation, with a particular focus on those employing connectivity analyze that enable inferences about directionality, *i.e*. the mechanisms by which regions influence each other. Findings for neutral and positive emotion are also included where relevant, to facilitate a more comprehensive understanding of emotion processing.

Functional connectivity refers to statistical methods for estimating indirectly inferring co-activation of brain regions from changes in the blood-oxygen-level-dependent (BOLD) responses (O'reilly et al., 2012). Correlational methods such as psychophysiological interaction (PPI) allow for the discovery of regions that co-activate in relation to a chosen region of interest, during an experimental task (Friston et al., 1997). DCM, by contrast, does not allow for the discovery of co-activating regions, but rather tests a series of probability models on General Linear Model activation maps to best determine the connectivity architecture of the included regions. DCM allows researchers to make claims regarding the direction of influence with regards to connectivity changes within a proposed network (Friston, 2011).

## 2 Methods

### 2.1 Protocol and registration

The current review was added to the PROSPERO register on 08/01/2018 (Registration Number: CRD42018084389).

### 2.2 Eligibility criteria

Studies were included if they (1) included quantitative analyze of functional Magnetic Resonance Imaging data, (2) were published between 1991 and the present (as prior to this year, functional MRI had not been invented). (3) tested a sample of healthy participants (aged 18 or over), (4) analyzed functional connectivity (using any method), or (5) employed emotional processing tasks portraying distinct categories of emotion (happiness, sadness, fear, anger, disgust, etc.). There was no minimum sample size for included studies. Studies were excluded if they (1) solely analyzed functional activation, or resting-state or structural connectivity data (2) used healthy participants solely as a comparison group for a sample with a specific disorder, as these analyze focused on differences between groups, or (3) used non-human participants. Resting-state studies were not included as, while there is evidence that they may provide greater reproducibility and signal to noise ratio (Fox and Greicius, 2010) they entail more uncertainty regarding the affective and cognitive processes being engaged.

### 2.3 Information sources and search

Studies were primarily identified by searching electronic databases (Ovid and Web of Science), with further searches completed in Google Scholar and PubMed, using the same search terms. The reference lists of relevant articles were also checked. Where conference presentations or trial registrations were found, authors were contacted to check for resulting peer-reviewed journal articles. Only studies available in English were included. The following search topics were combined:

1. Magnetic resonance OR FMRI OR MRI.
2. Connectivity OR connections OR coupling OR granger OR PPI OR DCM.
3. Healthy OR controls OR general OR undergraduate OR normal NOT schiz* NOT depres* NOT PTSD NOT Parkinson* NOT OCD NOT bipolar NOT stroke NOT disorder NOT illness NOT traum* NOT animal NOT rodent NOT primate NOT nonhuman.
4. Emot* OR affect* OR social OR prosocial OR anger OR happy OR threat OR fear OR neutral OR sad OR sadness OR anxiety OR anxious OR mood OR disgust.

An asterisk after a term matched all terms that begin or end with that root. The search was run on 19th March 2020. Additional searches were performed on 28th June 2020 and 1st April 2021 to find new articles published since this time.

Due to the relative paucity of effective connectivity studies, an additional search was conducted to look for such studies examining clinical populations that reported the results for the healthy controls. The following search terms were combined:

1. Magnetic resonance OR FMRI OR MRI.
2. Healthy OR controls OR general OR undergraduate OR normal.
3. Disorder or psychiatric or patient or schizophrenia or depression or anxiety.
4. Emot* OR affect* OR social OR prosocial OR anger OR happy OR threat OR fear OR neutral OR sad OR sadness OR anxiety OR anxious OR mood OR disgust.
5. Dynamic causal modeling or modeling or granger or DCM or structural equation modeling or SEM or effective connectivity.

A further 7 studies eligible for inclusion were identified through this search (Breakspear et al., 2015; Dima et al., 2015; Goulden et al., 2012; Sladky et al., 2015; Tak et al., 2021; Torrisi et al., 2013; Vai et al., 2015), and are reported in the results below.

### 2.4 Study selection

After screening titles and abstracts, full-texts were assessed for eligibility. The full-texts were screened separately by two authors, RU and ET. Any discrepancies in the authors’ findings were resolved through discussion. Additional relevant studies were identified via the reference lists of full texts that were screened.

**Fig. 1:**
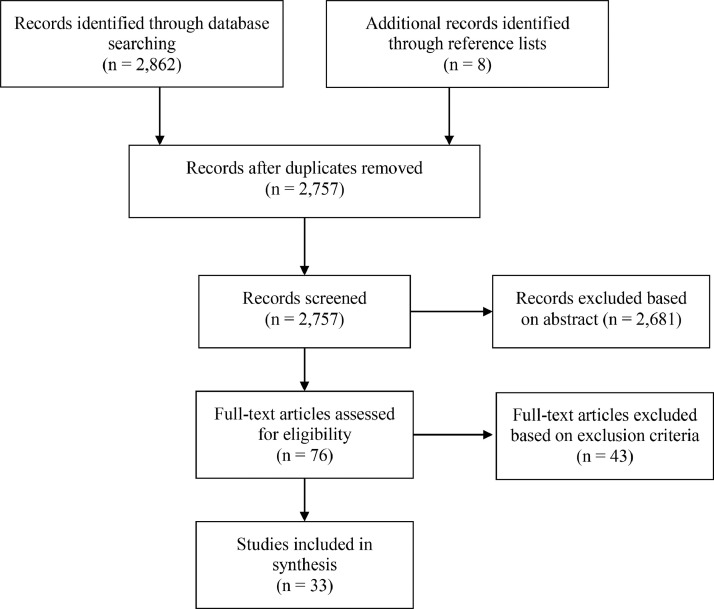
Flow chart depicting study inclusion.

Applying the inclusion and exclusion criteria, studies were excluded for the following reasons: if the sample did not include adults; if the sample did not include healthy control participants; if a control group was present but only between-group analyze were reported; if the study did not analyze functional connectivity; if the study did not measure BOLD-response data; if the study did not measure emotion processing (*e.g*. resting state connectivity); if they did not employ naturalistic stimuli such as facial emotions (*e.g*. the task stimulus was a primary reward); if the connectivity analysis was solely to investigate interactions between task conditions (*e.g*. a memory task with affective and non-affective task conditions); if only resting-state connectivity was measured, or periods of sustained emotional states (*e.g*. induced anxiety via threat of electric shock). Such studies were not deemed to be directly measuring the processing of other's emotions in a naturalistic context. Even in studies with multiple task conditions where the main effect of affect is reported, seed regions and dynamic causal models are typically driven by hypotheses relating to the processes of interest (*e.g*. memory), limiting their relevance to the present review. See Fig. 1 for the study inclusion flow chart.

### 2.5 Data extraction

The following details were extracted from each study: design, sample size, neuroimaging modality, experimental task used, connectivity measures and analyze. Currently there is no published assessment of quality and risk of bias tool for functional Magnetic Resonance Imaging (fMRI) studies. Therefore, a 10-item qualitative tool was developed for the current review. See supplementary materials for a description of the items included in the tool, alongside their development. Additionally, estimates of effect size (using Cohen's *d* for independent t-tests; (Cohen, 1988) were calculated where possible, using reported *Z*-scores, sample sizes, and degrees of freedom. Calculating Cohen's d for paired t-tests without using the original standard deviations for the two means produces overestimated effects (Dunlap et al., 1996). This would limit its usage in power calculations for future studies, for example. However, for the purpose of this review, the overestimation will be consistent across regions within studies. A Cohen's *d* of 0.2 or above indicates a small effect, *d* > 0.5 indicates a medium effect, and above 0.8 is considered a large effect.

### 2.6 Data and code availability

No neuroimaging data analyze were performed for this review.

## 3 Results

### 3.1 Structed assessment of quality

See supplementary table for tabulated quality ratings. All included studies presented their hypotheses and methodology clearly. Five studies did not indicate whether the stimuli employed were validated (Denny et al., 2014; Jabbi and Keysers, 2008; Mazzola et al., 2016; Miyahara et al., 2013; Tschacher et al., 2010). Furthermore, twelve studies did not assess the subjective valence of their stimuli i.e. through self-report (Breakspear et al., 2015; da Silva et al., 2010; de Marco et al., 2006; Fairhall and Ishai, 2006; Goulden et al., 2012; Hakamata et al., 2016; Jabbi and Keysers, 2008; Mazzola et al., 2016; Morawetz et al., 2017; Park et al., 2016; Payer et al., 2012; Vai et al., 2015). Neuroimaging data alone may not be sufficient to determine that the intended emotion (or level of arousal) has been evoked in participants. Apart from four studies (da Silva et al., 2010; Fairhall and Ishai, 2006; Jabbi and Keysers, 2008; Tschacher et al., 2010), the included papers adequately described the sample being studied. Less well-defined samples were missing information about participant demographics, as well as whether participants were screened for a history of psychiatric illness, meaning that the presence of clinical diagnoses cannot be excluded.

Ten studies defined the coordinates for their regions of interest using observed data (Blasi et al., 2009; Breakspear et al., 2015; Denny et al., 2014; Fairhall and Ishai, 2006; Fastenrath et al., 2014; Furl et al., 2013; Jabbi and Keysers, 2008; Morawetz et al., 2015; Schienle and Scharmuller, 2013; Sripada et al., 2014). In the majority of studies therefore, ROIs were defined a priori using anatomical masks. While this approach is common, and allows for greater comparability across studies, defining ROIs a priori may miss experiment-specific effects (Brooks et al., 2017).

Six studies presented data from samples whose size fell below 16 (Banks et al., 2007; de Marco et al., 2006; Fairhall and Ishai, 2006; Furl et al., 2013; Payer et al., 2012; Williams et al., 2006). The minimum sample size necessary to find reliable effects in fMRI data remains contentious (Button et al., 2013), as it depends on the design, with recommendations ranging from 16 (Friston, 2012) to 24 participants (Desmond and Glover, 2002). Given the range of recommended sample sizes, we took an inclusive approach and followed the minimum sample size of 16 proposed by Friston (2012). Additionally, only one study provided a justification for their sample size (Payer et al., 2012). Eleven studies did not apply statistical corrections for multiple comparisons (Banks et al., 2007; Das et al., 2005; de Marco et al., 2006; Goulden et al., 2012; Hakamata et al., 2016; Jabbi and Keysers, 2008; Payer et al., 2012; Tak et al., 2021; Torrisi et al., 2013; Tschacher et al., 2010; Vai et al., 2015), with three of these not providing a justification (de Marco et al., 2006; Jabbi and Keysers, 2008; Payer et al., 2012). The effect sizes calculated for the present review were typically very large across studies. These effects were likely inflated by studies reporting uncorrected scores and smaller samples. Although an overall grade cannot be provided for the quality of the included studies, it is worth highlighting that the majority presented appropriate methods and analyses that were adequately described by the authors.

### 3.2 Findings

**Fig. 2:**
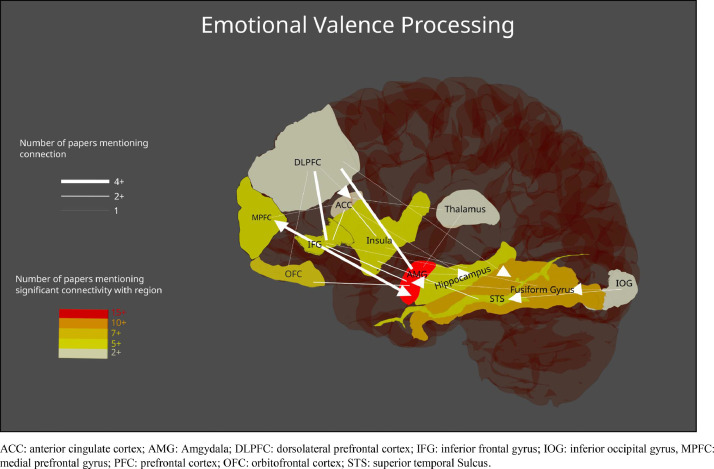
Regions most highly represented in the reviewed studies examining emotional valence processing. Thickness of lines represent number of studies finding a connection. Arrowheads represent direction of influence found in effective connectivity analyze.

**Fig. 3:**
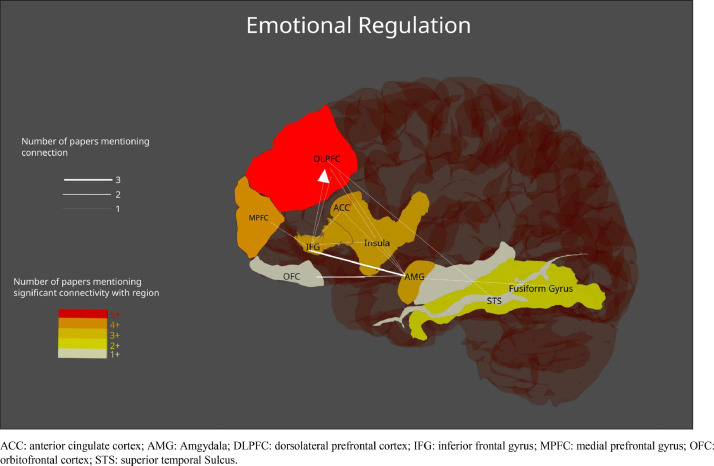
Regions most highly represented in the reviewed studies examining emotion regulation. Thickness of lines represent number of studies finding a connection. Arrowheads represent direction of influence found in effective connectivity analyze.

**Fig. 4:**
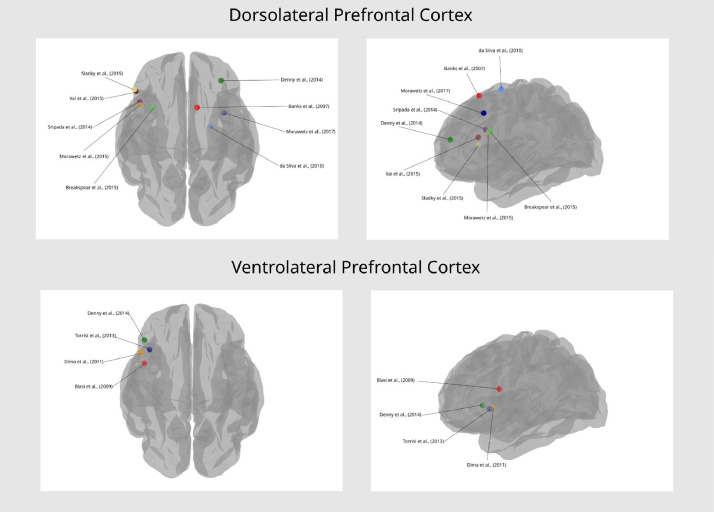
Coordinates reported in the connectivity findings of included studies under the anatomical labels ‘dorsolateral prefrontal cortex’ and ‘ventrolateral prefrontal cortex’.

**Fig. 5:**
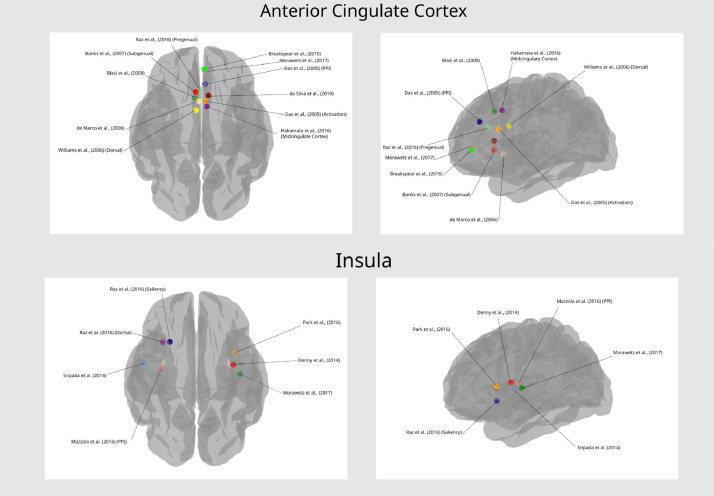
Coordinates reported in the connectivity findings of included studies under the anatomical labels ‘anterior cingulate cortex’ and ‘insula’.

The studies included in the current review represent a wide range of experimental tasks, designs, and processes of interest, including emotional salience, recognition, and regulation. To help extract generalities across studies, and avoid repetition of the table contents, findings have been grouped below under different subheadings. Studies employing methods for estimating effective connectivity have primarily been summarized under Section 3.3. See Figs. 2 and 3 for a visual representation of the regions most commonly reported in emotional valence processing and emotion regulation processing studies respectively (note the anatomical areas and labels were drawn from three-dimensional meshes produced for the software package Blender, sourced from structural atlases published by Desikan et al. (2006), Destrieux et al. (2010), and Klein and Tourville (2012); see https://brainder.org/research/brain-for-blender/ for further details). See Figs. 4 and 5 for plots of the coordinates reported across the included studies for larger anatomical regions, namely the dorsolateral prefrontal cortex, the ventrolateral prefrontal cortex, the anterior cingulate cortex, and the insula. MNI coordinates and Brodmann areas for these regions have also been added to Table 1.

**Table 1:** Summary of studies examining functional and effective connectivity in emotion processing in healthy participants.

Study	Analysis(sample size) - software	Task (contrast)	Seed(s) (constraint)	Statistical threshold	Findings (Brodmann Area, MNI coordinates; effect size)	Comment
Functional connectivity						
Banks et al. (2007)	PPI analysis (*n* = 14) – SPM2	Block design. Reappraisal vs maintain response to negative IAPS images (fixation cross)	AMG (a priori)	*P* < 0.05 small volume corrected*, *p* < 0.001 uncorrected	Reappraisal, relative to maintaining emotional response, led to increased connectivity between AMG, DLPFC (BA8, −12, 22, 60; *d* = 3.08), OFC (*d* = 3.01), sgACC (BA25, 6, 24, −2; *d* = 2.64), DMPFC (*d* = 2.50) and IPC (*d* = 2.12).	Stimuli taken from widely used image set. Relaxed statistical thresholding and small sample size. Normed stimulus valence ratings were reported.
Blasi et al. (2009)	PPI analysis (*n* = 43) – SPM5	Event-related implicit facial perception (sex discrimination) task using angry, fearful, and neutral faces (fixation cross)	AMG (a priori, but data driven)	FWE *p* < 0.05 small volume corrected	Fearful and angry faces were associated with increased activation in AMG, DLPFC, VMPFC, and VLPFC (BA44, 52, 12, 18). PPI of activation in response to neutral faces found increased AMG-ACC (BA32, 8, 24, 40; *d* = 0.91) connectivity.	Stimuli taken from widely used Nimstim facial set (Tottenham et al., 2009). Appropriate statistical thresholding and sample size. Detailed methodology. Behavioural measure used to check stimuli valence.
Das et al. (2005)	PPI analysis (*n* = 28) – SPM2	Block design. Explicit viewing of fearful faces (neutral faces)	AMG, ACC, thalamus, IOG, FG (a priori)	*P* < 0.05 small volume corrected*, *p* < 0.01 small volume corrected	Fearful faces increased activation in all ROIs bilaterally, relative to neutral faces. Decreased connectivity was observed between thalamus (*d* = 1.46), vACC (BA32, −4, 18, 20; *d* = 1.13), and amygdala. FG and IOG also showed negative connectivity with left amygdala, while the FG (*d* = 1.13) showed positive connectivity with the right amygdala only. Authors suggested hemispheric specialisation in processing regarding fear processing.	Stimuli previously developed for use with neuroimaging (Gur et al., 2002). Adequate sample size. Stimuli valence assessed post-scan. Relaxed statistical threshold, with novelty of study given as justification.
Denny et al. (2014)	PPI analysis (*n* = 25) - SPM8 for pre-processing, GLM in Neuroelf	Block design. Habituation task using negative EPS images (neutral IAPS images)	Insula (a priori, but data driven)	Whole-brain FWE *p* < 0.05*, FWE small volume corrected	Habituation led to decrease in AMG, occipital gyrus and VLPFC (BA47, 48, 33, −3) activity, whereas insula (BA48, −39, −3, −12), DLPFC (BA46 −36, 45, 15) and precuneus showed increased activation. Negative images were associated with increased connectivity between insula and AMG (*d* = 1.71), which correlated with behavioural habituation.	Stimuli drawn from validated image sets (EPS; Geday et al., 2001; IAPS; Lang et al., 1999). Adequate sample size and statistical thresholding. Behavioural valence check was reported. Habituation was examined using a single repetition of the stimulus.
Hakamata et al. (2016)	PPI analysis (*n* = 41) - SPM8	Event-related. Unattended fearful faces (neutral faces)	Pulvinar (a priori)	Cluster-level FWE *p* < 0.05*, *p* < 0.0001 uncorrected	Fearful faces showed increased AMG activation. Unattended fearful faces increased connectivity between pulvinar and AMG (*d* = 1.23), FG (*d* = 1.76), IFG (*d* = 1.76), anterior midcingulate cortex (BA32, −6, 14, 40; *d* = 1.76), SPL (*d* = 1.76), and SMA (*d* = 1.76), relative to unattended neutral faces.	Employed widely used Ekman faces. Adequate sample size and statistical thresholding. PPI analysis thresholding more liberal, but justified as exploratory. No behavioural measures used.
Hrybouski et al. (2016)	repeated-measures ANOVA using Fisher Z-transformed partial correlations (*n* = 25) - SPM8	Event-related. Emotional valence rating of high negative emotion IAPS pictures (neutral pictures)	AMG subdivided into centromedial, basal, and lateral nucleus groups (a priori)	FWE *p* < 0.05*, FWE *p* < 0.05	Centromedial AMG elicited most activation in response to negative, relative to neutral stimuli. All subnuclei groups showed significant connectivity with one another. Connectivity was strongest between lateral and basal AMG nuclei, converging with findings from animal studies.	Appropriate statistical thresholding and sufficient power. Stimuli piloted prior to the study and rated post-scan to check emotional valence. Image valence was tested behaviourally post-scan. Connectivity analysis limited to correlated activation.
Jabbi & Keysers (2008)	PPI & Granger causality analyze (*n* = 18) - SPM2	Event-related. Clips of actor sipping drink and reacting with disgust, happiness (neutral response)	IFO, IFG (a priori, data driven)	*P* < 0.05 uncorrected	Stronger activation in IFO following emotional vs neutral clips. IFO is causally triggered by activity in the IFG during emotional clips. Whole-brain PPI showed regional specificity of IFG – IFO connectivity.	Novel stimuli piloted in a previous study. Modest sample size. Relaxed statistical thresholding, which the authors do not justify. No behavioural valence check reported.
Miyahara et al. (2013)	PPI analysis (*n* = 18) - SPM8	Event-related. Explicitly attended threatening faces of male convicted murderers (neutral faces)	AMG, FG, STS, ITG (a priori)	FWE *p* < 0.05	AMG activation did not differ between murderers’ faces and neutral faces. Connectivity between AMG, STS (*d* = 2.37), ITG (*d* = 2.05) and FG (*d* = 2.23) was correlated with subjective threat rating of faces.	Control faces taken from widely used Centre for Vital Longevity Face Database. Modest sample size, but appropriate statistical thresholding. Novel stimuli used, which were checked for threat valence behaviourally. However, photos did not represent specific emotional expressions (i.e. anger/fear) or intensities.
Morawetz et al. (2017)	PPI analysis (*n* = 23) – not reported	Event-related emotional regulation (increase & decrease) of aversive IAPS images (neutral images)	IFG, AMG (a priori)	FWE *p* < 0.05*, FWE *p* < 0.05	Successful down-regulation of emotion correlated with increased connectivity between IFG, DLPFC (BA9, −43, 13, 39), DMPFC, MTG and STG, as well as negatively correlated connectivity between IFG and VMPFC. Successful up-regulation of emotion correlated with increased connectivity between IFG, STG (*d* = 0.25), and AMG (*d* = 0.25), as well as negatively correlated connectivity between IFG, pgACC (BA32, −12, 38, 10; *d* = 0.35), DMPFC (*d* = 0.26), caudate (*d* = 0.30), insula (BA48, −45, −13, 7; *d* = 0.22), IPL (*d* = 0.24), and MFG (*d* = 0.29). Successful up-regulation led to increased connectivity between AMG, IFG (*d* = 0.30), MFG (*d* = 0.30), ACC (*d* = 0.25), STG (*d* = 0.27), insula (*d* = 0.27), IPL (*d* = 0.27), MTG (*d* = 0.26), and parahippocampal gyrus (*d* = 0.22), as well as negatively correlated activity between AMG and OFC (*d* = 0.28). Successful down-regulation led to increased connectivity between AMG, FG (*d* = 0.27), and parahippocampal gyrus (*d* = 0.24).	Stimuli taken from widely used image set. Participants received training session in emotion reappraisal strategies. Appropriate statistical thresholding and modest sample size. No behavioural measure to check stimulus valence.
Park et al. (2016)	Group-wise graph-theoretical analysis (*n* = 19) - SPM8	Block design. 1-back task using negative and neutral faces (fixation cross)	AMG, FG, IFG, IOG, STS, VLPFC (a priori)	FWE *p* < 0.05*, *p* < 0.05 uncorrected at ROI-level	Negative faces increased activation in AMG and insula (BA45, −40, 10, 8). Global efficiency was higher within emotion processing areas (AMG, IFG, and OFC) and lower between visual (IOG, FG, and STS) and emotion processing areas for negative relative to neutral faces.	Stimuli less widely used but more ecologically valid (Korean face images and participants). Appropriate statistical thresholding. Modest sample size. No behavioural measure of stimuli valence.
Payer et al. (2012)	PPI analysis (*n* = 10) – ROIs in FSL, GLM in SPM8	Event-related emotion regulation task using aversive IAPS images (neutral images), instruction to ‘decrease’ or ‘look’. Block designed task either matching or labelling angry, scared, happy, surprised faces (shapes).	AMG, IFG (a priori)	*P* < 0.005 uncorrected	During reappraisal, AMG activation decreased with emotional intensity. AMG activation also decreased during affect labelling, when compared to matching. Emotion regulation led to increased AMG-IFG connectivity, as did affect labelling (rather than matching). When compared to the labelling task, emotion regulation showed increased AMG-IFG (*d* = 2.29) connectivity.	Stimuli taken from widely used IAPS and Nimstim image sets. Small sample size. Statistical thresholding for activation was not stated, and threshold used for PPI not corrected for multiple comparisons, without justification. No behavioural measure of stimuli valence in emotion regulation task.
Raz et al. (2016)	Network cohesion index analysis (*n* = 203) – not reported	Event-related passive viewing of film clips evoking sadness, fear, anger (black screen)	Domain-general networks including OFC, ACC, AMG, insula, hippocampus FG, putamen, and temporal pole (a priori)	whole-brain FDR correction (qFDR < 0.05)	Activation data was not reported. Emotional intensity was associated with stronger connectivity between the dorsal salience network (insula (BA1/13, 35, 20, 4), pgACC (BA32, 3, 30, 23), IFG, SMG) and the medial amygdala network (AMG, hippocampus, nucleus accumbens, VMPFC, sgACC (BA32, 2, 27, 1), temporal pole). Emotional intensity was also associated with stronger connectivity between the dorsal salience network and the ventrolateral amygdala network (AMG, temporal pole, STS, FG, OFC).	High power owing to large sample size, and appropriate statistical thresholding. Used stimuli valence measure post-scan.
Satterthwaite et al. (2011)	PPI analysis (*n* = 44) - FSL	Emotion identification task using neutral, happy, sad, angry, and fearful faces (crosshair matching face's perceptual qualities)	AMG, VSTR (a priori)	Monte Carlo simulation *p* < 0.05	Increased AMG, OFC, STS and IFG activation for threatening (angry or fearful), relative to non-threat (sad or happy) faces. No difference in VSTR activation between conditions. An overall connectivity analysis found decreased connectivity between AMG and VSTR. A PPI analysis found increased connectivity between AMG and OFC (*d* = 1.67) during threat vs non-threat condition. A second PPI with VSTR as seed found increased connectivity between VSTR and the parahippocampal gyrus (*d* = 1.14) during non-threat, relative to the threat condition.	Used validated stimuli generated for a previous study. Appropriate sample size and statistical thresholding. Stimuli valence was measured behaviourally. Image acquisition excluded dorsal regions.
Schienle & Scharmuller (2013)	PPI analysis (*n* = 34) - SPM8	Event-related. Passive viewing of disgust, happiness, and neutral images (fixation cross)	Insula, AMG, OFC, DLPFC, VLPFC, putamen, cerebellum, vermis (peak activation)	FWE *p* < 0.05*, FWE *p* < 0.05	Relative to neutral images, disgust and happiness led to increased cerebellum and vermis activation. Relative to neutral images, disgust increased cerebellum-vermis connectivity (*d* = 1.60), while happiness increased cerebellum-occipital gyrus connectivity (*d* = 2.61). When contrasted with each other, disgust and happiness increased cerebellum-AMG connectivity, albeit in the right cerebellar hemisphere for happiness (*d* = 1.21), and the left for disgust (*d* = 1.70). Happiness, relative to disgust, increased cerebellum-OFC and putamen connectivity.	Used validated stimuli generated for a previous study. Appropriate sample size and statistical thresholding. Stimuli valence was measured. Only recruited female participants, as authors’ previous study found greater disgust sensitivity in women.
Sripada et al. (2014)	PPI & Network contingency analysis (*n* = 49) - SPM8	Block design, using aversive IAPS images. Participants either maintained or reappraised emotional response. Participants taught reappraisal strategies prior to scan. (viewing images without instruction).	Intrinsic connectivity networks including visual, dorsal attention, frontoparietal, and default networks. Total of 837 ROIs (connectomic PPI)	*P* < 0.001 uncorrected*, *p* < 0.001 uncorrected *q* < 0.05 FDR-corrected for comparing PPI analyze	When contrasting reappraisal with the maintain condition, activation increased in dlPFC (BA45, 54, 24, 28), dmPFC, SPL caudate, and STS, and reduced activity in insula (BA13, 54, −2, 0) and rolandic operculum. Relative to maintaining emotional response, reappraisal produced changes in functional connections across all networks. Visual network in particular increased connectivity with dorsal attention and default networks. Authors take this to mean these networks mediate critical processes such as visual processing, stimulus salience, attentional control, and interpretation and contextualization of stimuli.	Widely used stimulus set. Appropriate sample size. Participants’ affective state was measured behaviourally. Appropriate statistical thresholding for the network contingency analysis, but looser thresholding for activation and PPI analyze, which the authors justify as valid for connectomic PPI.
Williams et al. (2006)	PPI analysis (*n* = 15) - SPM2	Conscious and non-conscious face perception task using block design. Fearful faces (neutral faces)	AMG, brainstem, thalamus, striate visual cortex, FG, IOG, ACC, OFC, SFG (a priori)	small volume corrected *p* < 0.05	Conscious fear elicited activation in AMG, thalamus, striate cortex, IOG, FG, and ACC. Nonconscious fear elicited activation in AMG, thalamus, brainstem, and to a lesser degree in VMPFC. PPI analysis of conscious fear found negative connectivity between AMG, thalamus (*d* = 1.81), striate cortex (*d* = 1.83), and brainstem (*d* = 1.50), but positive connectivity between AMG and dACC (BA24/33, 6, 8, 24; *d* = 1.80). Nonconscious fear found positive connectivity between AMG, brainstem (*d* = 1.33), thalamus (*d* = 1.72), midbrain (*d* = 1.55), rACC (*d* = 1.57), and MPFC (*d* = 1.50), and negative connectivity between AMG, VMPFC (*d* = 2.14) and, IOG (*d* = 1.87).	Stimuli previously developed for use with neuroimaging (Gur et al., 2002). Image valence was tested behaviourally post-scan, as was non-awareness during non-conscious trials. Small sample size, and relatively relaxed statistical thresholding, which the authors justify by a stringent random effects model with a priori defined regions.
Effective connectivity						
Breakspear et al. (2015)	DCM analysis (*n* = 45) - SPM	Event-related. Go/no-go task with fearful, happy, and calm faces (gender discrimination task)	FG, ACC, DLPFC, IFG (data driven)	Whole-brain FWE *p* < 0.05*	Network nodes derived from whole-brain activation. 8 models were specified, each with direct input to the FG, a forward connection from FG to ACC (BA10, −3, 47, −2) modulated by fear, and FG to DLPFC (BA48, −39, 17, 25) modulated by motor inhibition. The models varied how these modulatory influences converged on the IFG. Model selection favoured non-linear models in which the modulatory effects of fear and motor inhibition on the IFG gated each other in a dynamic, non-hierarchical way.	Widely used Nimstim faces set. Adequate sample size, and appropriate statistical thresholding. No behavioural valence check
da Silva et al. (2010)	DCM analysis (*n* = 17) - SPM8	Event-related. Passive viewing of low/high sadness and neutral faces (fixation cross)	ACC, DMPFC, DLPFC, FG (a priori)	Whole-brain FWE *p* < 0.05*	All faces elicited activation in FG, ACC (BA32, −7, 22, 8), DLPFC (BA6/9, 22, 19, 56; −30, −2, 63) and DMPFC. A DCM was specified with bidirectional endogenous connections between all regions, and direct input of emotion to the FG. An increase in sadness led to increased connectivity between prefrontal areas and ACC with FG. There was also greater modulation of connectivity in ACC and FG by prefrontal areas.	Employed widely used Ekman et al. faces (1975). Modest sample size, appropriate thresholding. A priori ROIs driven by Parkinson's Disease literature, limiting generalisability. No stimuli valence check.
de Marco et al. (2006)	SEM (*n* = 10 & 14) - SPM99, SEM in AMOS	Gender & emotion identification tasks. Block design. Fearful and ambiguous faces (neutral faces)	AMG, ACC, OFC (a priori)	*P* < 0.05 uncorrected*	Both tasks increased activation in all ROIs bilaterally. Path coefficients analysis models were created for each hemisphere. Results in left hemisphere suggested fear pathway from AMG to OFC via ACC (BA24/25, 3, 14, −6) during implicit perception, while explicit perception found reversed route from OFC to ACC, with weak AMG-ACC connectivity. AMG-OFC connectivity was weak across tasks. Similar results were found in right hemisphere, but below significance threshold.	Employed widely used Ekman faces. Small samples and relaxed statistical thresholding, neither of which were justified. Difference in sample sizes between experiments is unexplained. No stimuli valence check was performed.
Dima et al. (2011)	DCM analysis (*n* = 40) - SPM8	Event-related affect recognition task using angry, fearful, and sad faces (fixation cross)	AMG, FG, IOG, VLPFC (a priori)	Whole-brain FWE *p* < 0.05*	Compared to neutral faces, emotional faces elicited increased activation in occipital areas including IOG and FG, frontal areas including IFG and MFG, the STG, and the AMG. The specified models contained bidirectional endogenous connections between all regions, with direct input to the IOG. Of 11 models, the winning model had affect modulating forward connections to the VLPFC (BA47, 51, 20, −6). All faces led to increased effective connectivity from IOG to VLPFC. Modulation of connections towards VLPFC were not mediated by AMG.	Stimuli drawn from widely used Ekman faces. Stimuli valence check was used. Adequate sample size and appropriate statistical thresholding.
Dima et al. (2015)	DCM analysis (*n* = 46) – SPM8	Event-related. Affect recognition task using fearful, angry or sad faces (neutral faces)	IOG, FG, AMG, VPFC (a priori)	Whole-brain FWE *p* < 0.05*	The specified model contained the driving input to the IOG, and bidirectional endogenous connections between all nodes, apart from between the FG and AMG. Across 7 models, the optimal model had negative faces increase the strength of the forward connection from the IOG to the VPFC.	Widely used Ekman faces. Adequate sample size and appropriate statistical thresholding. Task incorporated behavioural valence check.
Fairhall & Ishai (2006)	DCM analysis (*n* = 10) - SPM5	Event-related attentive viewing of famous and unfamiliar fearful and happy faces (scrambled faces)	AMG, FG, IFG, IOG, OFC, STS (a priori, data driven)	*P* < 0.001 uncorrected*	Activation increased in all ROIs during all faces, relative to scrambled ones. Created models representing core (IOG, FG, STS) and extended (AMG, IFG, OFC) systems. Winning core model had IOG separately influencing FG and STS; subsequent analysis compared models with feedforward connection from FG or STS to extended system. All faces led activation to feed forward from the IOG to the FG and STS in the core system, with the FG only feeding forward to the extended system. Emotional faces increased effective connectivity from the FG to the AMG.	No demographic or clinical information reported. Stimuli design not specified. No behavioural test of stimuli valence reported. Small sample size, and moderately relaxed statistical thresholding, neither of which were justified by authors.
Fastenrath et al. (2014)	DCM analysis (*n* = 574) - SPM8, Bayesian Model Averaging in R	Event-related. Pleasant, neutral and aversive IAPS images that were used in an encoding task (scrambled images)	AMG, hippocampus (data driven)	Small volume Bonferroni-corrected *P* < 0.05*	Peak activation in AMG and hippocampus was used to select ROIs. 192 models varied direct and modulatory inputs for connections between both regions. Winning DCM model indicated external input from stimuli to AMG but not hippocampus. Connectivity projecting from AMG to hippocampus increased in strength during encoding of both positive and negative images, relative to neutral ones.	Stimuli drawn from widely used IAPS set. High statistical power owing to large sample size. Bonferroni correction appropriate given inflated type I error. Image valence was tested behaviourally post-scan.
Furl et al. (2013)	DCM analysis of fear sensitivity (*n* = 13) - SPM8	Block design. Passive viewing of dynamic & static disgusted, happy, and fearful faces (scrambled faces)	AMG (a priori), OFA, FG, MTVA, STS, (peak activation)	Cluster-level FWE *p* < 0.05*	All Static and dynamic faces activated AMG, relative to scrambled faces. The FG showed increased sensitivity to static fearful vs non-fearful faces, but not when faces were dynamic. Specified models varied the following: direct input or not to AMG, all combinations of modulatory effects of dynamic and static faces, and full or feedforward endogenous connectivity. Of 508 models, winning DCM model found AMG mediates fear sensitivity in visual areas and mode of presentation (i.e., dynamic vs static faces) determines regional top-down effect. All ROIs had connectivity, exogenous inputs only to OFA and MTVA. Connections from AMG to STS and MTVA modulated by dynamic fear. AMG to FG and FG to MTVA connections modulated by static fear.	Stimuli from recently validated facial image set. Used localiser runs to identify ROIs. Small sample size, but appropriate statistical thresholding. Image valence was tested behaviourally post-scan.
Goulden et al. (2012)	DCM analysis (*n* = 21) – SPM8	Block design. Passive viewing of happy, sad, fearful faces, neutral faces (Rest)	IOG, FG, AMG, OFC (a priori)	*P* < 0.05 uncorrected*	Contrasting all faces with rest elicited activation in pre-specified ROIs. Specified models all had direct input to the IOG, feeding forward to the FG, and intrinsic bidirectional connections between the FG, AMG, and OFC. Each model varied location and direction of modulatory effects on connections between FG, AMG, and OFC. In winning models, sad faces modulated bi-directional connections between AMG and OFC and between FG and OFC; happy faces modulated unidirectional connections from FG to OFC.	Employed widely used Ekman faces. Modest sample size, relaxed statistical thresholding. No behavioural check for stimuli valence.
Mazzola et al. (2016)	PPI and DCM analyze (*n* = 20) - SPM8	Event-related. Clips of person grasping objects with angry or joyful expressions (neutral expression)	Insula and STG (a priori)	FWE *p* < 0.05*, whole-brain FWE *p* < 0.01	Contrasting faces vs. grasping alone only elicited activation in temporal gyrus. Greater activation in insula (BA13, 38, −7, 10) during angry expression, relative to the joyful one. Both analyze showed changes in connectivity during the angry situation. 14 models varied modulatory inputs and endogenous connections between insula, right STG and left STG, with direct input to insula. DCM analysis showed anger increased forward connection from right insula to right STG & suppressed forward connection from right insula to left STG.	Novel stimuli piloted in a previous study. Modest sample size, and appropriate statistical thresholding. No behavioural check for stimuli valence.
Morawetz et al. (2015)	DCM analysis (*n* = 23) - SPM8	Event-related emotion regulation task using extreme sports clips (neutral videos)	IFG, DLPFC, SMA, SMG (peak activation)	FWE *p* < 0.05*	During exposure to clips, activity increased in multiple regions including MTG, SMG, IFG, posterior cingulate and FG. During emotion regulation, activity increased in SMA, SMG, IFG, and DLPFC. In the DCM analysis, two families (9 models with DLPFC as central node, 9 with IFG) varied modulatory inputs. Winning model had DLPFC as central node of prefrontal emotion regulation network, strongly interconnected with IFG. During reappraisal, IFG effectively inhibited DLPFC.	Modest sample size and appropriate statistical thresholding. Behavioural measures use to assess stimuli valence and emotion regulation.
Sladky et al. (2015)	DCM analysis (*n* = 13) – SPM8	Block design. Facial emotion valence matching task using anger, disgust, fear, happiness, sadness, surprise, and calmness (object matching)	AMG, OFC, DLPFC (a priori)	FWE *p* < 0.05*	Activation was found in specified ROIs. Initial model contained direct input to AMG and DLPFC (BA45, 56, 34, 8). 128 models varied possible intrinsic and modulatory connections. Winning model found bidirectional intrinsic connections between AMG and OFC, with forward connections from AMG and OFC to DLPFC. Combined faces negatively modulated backward connection from OFC to AMG.	Widely used Nimstim set. Small sample size, but appropriate statistical thresholding. Task included behavioural valence check.
Tak et al. (2021)	DCM analysis (*n* = 28) – SPM12	Event-related. Passive viewing of negative IAPS images (neutral images)	IOG, PHG, OFC (a priori)	*P* < 0.001 uncorrected*	Activation was found in the specified ROIs. The specified model had full endogenous connectivity between all regions, and direct input of negative and neutral images to the IOG. 64 models varied which connections received modulatory input. The winning model had negative emotion modulating all connections. Specifically, excitatory influence by connections from the IOG to PHG and OFC, inhibitory influence by connections from the PHG and OFC to the IOG, and inhibitory influence by the connection from the OFC to the PHG.	Widely used IAPS image set. Modest sample size, and relaxed statistical thresholding. No behavioural valence check.
Torrisi et al. (2013)	DCM analysis (*n* = 45) – SPM8	Block design. Facial affect labelling and matching tasks using neutral, fearful, surprised, or angry expressions (matching forms)	IOG, AMG, VLPFC, Broca's area (a priori)	*P* < 0.05 uncorrected*	Activation was found in the specified ROIs. 64 models were estimated, with the winning model containing endogenous connections between Broca's area, the VLPFC (BA47, 42, 23, −7), and the AMG, with direct input to the IOG and forward connections from the IOG to AMG and VLPFC. Broca's area and VLPFC exerted a dampening influence on the AMG during affect labelling.	Widely used Ekman faces. Adequate sample size, and relaxed statistical thresholding. Task included behavioural valence check.
Tschacher et al. (2010)	Granger causality analysis (*n* = 20) – Vector auto regression in SAS	Block design. Implicit emotion processing task using audio clips of crying and laughter. Participants monitored pitch change (time-reversed clips)	AMG, insula, auditory cortex (a priori)	*P* < 0.05 uncorrected	Reversed audio clips did not elicit significant insula activation, relative to clips played forwards. Positive connectivity was found between all ROIs. However, activation in the left auditory cortex preceded activity in the right amygdala, which authors interpreted as active inhibition of amygdala by cortical regions.	Stimuli valence measured behaviourally. Modest sample size, and relaxed statistical thresholding, justified by exploratory nature of study. Novel stimuli created for study.
Vai et al. (2015)	DCM analysis (*n* = 29) – SPM8	Block design. Face-matching task using fearful and angry expressions (geometric shapes)	IOG, AMG, FG, DLPFC, VPFC (a priori)	*P* < 0.001 uncorrected*	Activation was found in specified ROIs. 48 models were compared. The winning model had direct input to the IOG, FG and AMG; bidirectional endogenous connections between DLPFC (BA47, 56, 34, 18), VPFC and AMG, forward connections from VPFC to DLPFC, IOG to FG, and FG to AMG. Faces modulated bidirectional connections between AMG and DLPFC, and the connection from VPFC to AMG.	Widely used Ekman faces. Modest sample size, relaxed statistical threshold. No behavioural valence check.
Willinger et al. (2019)	DCM analysis (*n* = 33) - SPM12	Face-matching task using dynamic happy, surprised, sad, disgusted, and neutral faces (shape-matching task)	AMG, MPFC, LPFC, FG (a priori, data driven)	FWE *p* < 0.05*	Activation was found in regions composing intended DCMs, as well as STG, cerebellum, and parahippocampal gyrus. 256 models with bidirectional endogenous connectivity, grouped into four families, varied modulatory inputs. Winning model found effective connectivity from MPFC to AMG was modulated by positive and negative valence. Bottom-up connectivity from the AMY to the MPFC was modulated by negative and neutral, but not positive valence. Connection from LPFC to MPFC was modulated by negative and positive valence.	Stimuli with good ecological validity. Stimuli valence measured behaviourally. Adequate sample size, appropriate statistical thresholding.

D: dorsal; V: ventral; M: medial; L: lateral; R: rostral; PG: pregenual; SG: subgenual; ACC: anterior cingulate cortex; dACC: dorsal anterior cingulate cortex; vACC: ventral anterior cingulate cortex; AMG: amgydala; DCM: dynamic causal modelling; DLPFC: dorsolateral prefrontal cortex; DMPFC: dorsomedial prefrontal cortex; EPS: Empathy Picture System; FG: fusiform gyrus; FDR: false discovery rate; FSL: FMRIB Software Library; FWE: family-wise error corrected; IAPS: international affective picture system (Lang and Bradley, 2007); IFG: inferior frontal gyrus; IFO: anterior insula and adjacent frontal operculum; IOG: inferior occipital gyrus, IPC: inferior parietal cortex; IPS: intraparietal sulcus; ITG: inferior temporal gyrus; MEG: Magnetoencephalography; MFG: middle frontal gyrus; MTG: middle temporal gyrus; MTVA: middle temporal visual area; OFA: occipital face area; OFC: orbitofrontal cortex; PFC: prefrontal cortex; PHG: parahippocampal gyrus PPI: psychophysiological interactions; ROI: region of interest; SEM: structural equation modelling; SMA: supplementary motor area; SMG: supramarginal gyrus; SPL: superior parietal lobule; SPM: statistical parametric mapping; STG: superior temporal gyrus; STS: superior temporal Sulcus; VPFC: ventral prefrontal cortex; VLPFC: ventrolateral prefrontal cortex; VMPFC: ventromedial prefrontal cortex; VSTR: ventral striatum. ‘Connectivity’ refers to positive connectivity unless otherwise specified. *statistical thresholding method used was for activation analysis only.

#### 3.2.1 Studies employing emotional valence processing tasks

Twenty seven studies employed tasks that required participants to implicitly or explicitly process the emotional valence of stimuli (Blasi et al., 2009; Breakspear et al., 2015; da Silva et al., 2010; Das et al., 2005; de Marco et al., 2006; Dima et al., 2011, 2015; Fairhall and Ishai, 2006; Fasternath et al., 2014; Furl et al., 2013; Goulden et al., 2012; Hakamata et al., 2016; Hrybouski et al., 2016; Jabbi and Keysers, 2008; Mazzola et al., 2016; Miyahara et al., 2013; Park et al., 2016; Raz et al., 2016; Satterthwaite et al., 2011; Schienle and Scharmuller, 2013; Sladky et al., 2015; Tak et al., 2021; Torrisi et al., 2013; Tschacher et al., 2010; Vai et al., 2015; Williams et al., 2006; Willinger et al., 2019).Twenty of these studies used faces to investigate emotion processing (Blasi et al., 2009; Breakspear et al., 2015; da Silva et al., 2010; Das et al., 2005; de Marco et al., 2006; Dima et al., 2015, 2011; Fairhall and Ishai, 2006; Furl et al., 2013; Goulden et al., 2012; Hakamata et al., 2016; Jabbi and Keysers, 2008; Mazzola et al., 2016; Miyahara et al., 2013; Park et al., 2016; Satterthwaite et al., 2011; Sladky et al., 2015; Torrisi et al., 2013; Vai et al., 2015; Williams et al., 2006).

##### 3.2.1.1 The amygdala

The amygdala was a frequently selected seed region for psychophysiological interaction (PPI) analyze, structural equation modeling, graph-theoretical analysis, and dynamic causal modeling (Blasi et al., 2009; Das et al., 2005; de Marco et al., 2006; Dima et al., 2015, 2011; Furl et al., 2013; Goulden et al., 2012; Miyahara et al., 2013; Park et al., 2016; Satterthwaite et al., 2011; Sladky et al., 2015; Torrisi et al., 2013; Vai et al., 2015; Williams et al., 2006). Changes in activation of amygdala and its connectivity to other regions were not limited to any facial emotion, including neutral ones. This was also true across task demands. For example, Blasi et al. (2009) found that connectivity increased between the amygdala and the anterior cingulate cortex (ACC) during implicit perception of neutral faces, relative to a fixation cross. The authors interpreted this as indicating the amygdala's role in processing ambiguous social stimuli.

However, Satterthwaite et al. (2011) found increased connectivity between the amygdala and the orbitofrontal cortex while participants identified angry and fearful faces explicitly, relative to sad and happy faces. Furthermore, Williams et al. (2006) found increased connectivity between the amygdala and the ACC during consciously perceived fearful faces, as well as during non-consciously perceived fearful faces, relative to neutral ones.

##### 3.2.1.2 Connectivity with other regions

The amygdala showed changes in connectivity with surrounding regions, particularly frontal areas such as the ACC and the ventromedial, medial, and dorsolateral PFC (Banks et al., 2007; Blasi et al., 2009; Breakspear et al., 2015; de Marco et al., 2006; Fairhall and Ishai, 2006; Morawetz et al., 2017; Park et al., 2016; Payer et al., 2012; Sladky et al., 2015; Vai et al., 2015; Willinger et al., 2019). Of course, changes in connectivity were also observed between the amygdala and other regions such as the thalamus and insula, as well as visual areas including the fusiform gyrus, the inferior occipital gyrus, and the superior temporal sulcus (Denny et al., 2014; Dima et al., 2015, 2011; Furl et al., 2013; Goulden et al., 2012; Torrisi et al., 2013; Tschacher et al., 2010; Vai et al., 2015; Williams et al., 2006). See Figs. 2 and 3 for a visual summary of connectivity between the regions most highly represented in the findings.

##### 3.2.1.3 Variation in connectivity strength

There was also variation as to whether connectivity increased or decreased between the amygdala and other regions, even when studies employed similar tasks. For example, Das et al. (2005) found decreased connectivity between the ventral ACC, the thalamus, and the amygdala while participants explicitly viewed fearful faces, relative to neutral ones. Williams et al. (2006) found that consciously viewed fearful faces also elicited negative connectivity between the amygdala and the thalamus, but positive connectivity between the amygdala and the ACC. These contrasting findings may speak to the complex nature of functional connectivity during emotion processing, as much as the variation in design between studies even when employing comparable tasks. Although both studies used fearful faces, their designs varied in other ways, including the imaging protocols, experimental procedures, and stimulus sets employed.

##### 3.2.1.4 Other regions of interest

It is also worth noting the variation in the cognitive processes and regions of interest across some of the included studies employing facial emotions. One study focused on the ‘gustatory cortex’, analyzing neural responses to actors filmed drinking a liquid and then reacting with happiness, disgust, or a neutral expression (Jabbi and Keysers, 2008). The authors concluded that in their experiment, activation in the inferior frontal gyrus (IFG) represented a mirroring of the motor function involved in facial expressions, while activity in the anterior insula and adjacent frontal operculum represented an empathic mirroring of the emotional state. However, the authors could not explain why connectivity between these regions strengthened during emotional clips, and that this was seemingly driven by activation in the IFG. The authors speculated that this may show that social emotion perception may be served by physical state perception, as the observer first processes the actor's facial movement, which then triggers the processing of the emotional state implied by their expression.

Hakamata et al. (2016) focused on the pulvinar as an a priori region of interest. The authors were interested in the relationship between selective attention and emotional processing, finding that unattended fearful faces increased connectivity between the pulvinar and the amygdala, fusiform gyrus, IFG, ACC, superior parietal lobule, and supplementary motor area. The authors explained that the pulvinar is not typically considered to have a significant role in emotion processing but may aid in the automatic attentional processing of threat. A dot-probe task conducted in the same study found that an attentional bias towards threat correlated with increased connectivity between the pulvinar and frontal regions, while participants with greater attentional control showed increased connectivity between the pulvinar and the amygdala.

Finally, one study employed a novel stimulus set, using real photos of males convicted of first-degree murder to represent threat (Miyahara et al., 2013). Each photo was rescaled to a common size and resolution. While such stimuli arguably provide greater ecological validity, the authors conceded that the photos did not represent a specific emotion or emotional intensity, especially since participants were not told the photos were of convicted murderers. Nonetheless participants rated them as significantly more threatening than images of neutral faces. Furthermore, threat ratings correlated with increased connectivity between the amygdala and other regions involved in face processing such as the superior temporal sulcus and the fusiform gyrus.

#### 3.2.2 Other emotion-inducing tasks

Twelve studies did not use facial expressions (Banks et al., 2007; Denny et al., 2014; Fastenrath et al., 2014; Hrybouski et al., 2016; Morawetz et al., 2017, 2015; Payer et al., 2012; Raz et al., 2016; Schienle and Scharmuller, 2013; Sripada et al., 2014; Tak et al., 2021; Tschacher et al., 2010). Image sets such as the International Affective Picture System (IAPS) were most commonly employed (Banks et al., 2007; Denny et al., 2014; Fastenrath et al., 2014; Hrybouski et al., 2016; Morawetz et al., 2017; Payer et al., 2012; Schienle and Scharmuller, 2013; Sripada et al., 2014; Tak et al., 2021). Similar to studies employing facial emotion tasks, the amygdala was frequently selected as a seed region.

One study carried out a network cohesion index analysis (Raz et al., 2016), which can quantify interactions within and between large networks, rather than changes in connectivity between individual regions (Raz et al., 2012). The authors examined responses to clips from popular films designed to evoke a range of negative emotions (sadness, fear, and anger) in several networks. These networks included two ‘salience’ networks and three amygdala-based networks associated with social affiliation and perception, and aversive responses. They found that as emotional intensity increased, the dorsal salience network, which includes the insula, ACC, IFG, and supramarginal gyrus (SMG), exhibited stronger connectivity with the medial amygdala network, which includes the hippocampus, the ACC, and the ventromedial prefrontal cortex (VMPFC). This interaction occurred regardless of the emotion being evoked, relative to a black screen.

#### 3.2.2 Studies employing emotion regulation tasks

While the majority of studies employed emotional valence processing tasks (*e.g*. passive viewing of angry faces), six studies used emotion regulation tasks (Banks et al., 2007; Denny et al., 2014; Morawetz et al., 2015; Morawetz et al., 2017; Payer et al., 2012; Sripada et al., 2014). As with the valence processing studies, the amygdala was a well-studied region, and despite the different task demands, reported similar findings. For example, Payer et al. (2012) found that both affect labeling and regulation in response to aversive images led to increased connectivity between the amygdala and the inferior frontal gyrus (IFG), relative to affect matching. Separately, Denny et al. (2014) found increased connectivity between the amygdala and insula correlated with habituation to negative images in participants. Both Banks et al. (2007) and Morawetz et al. (2017) found increased connectivity between the amygdala and prefrontal regions including the DLPFC and IFG during emotion regulation tasks involving negative IAPS images.

### 3.3 Dynamic causal modeling studies

Fifteen studies employed DCM (Breakspear et al., 2015; da Silva et al., 2010; Dima et al., 2015, 2011; Fairhall and Ishai, 2006; Fastenrath et al., 2014; Furl et al., 2013; Goulden et al., 2012; Mazzola et al., 2016; Morawetz et al., 2015; Sladky et al., 2015; Tak et al., 2021; Torrisi et al., 2013; Vai et al., 2015; Willinger et al., 2019). See Figs. 2 and 3 for an illustration of the directional relationships between the most highly represented regions. Each study employed models composed of an assembly of frontal, subcortical, and occipital regions, such as the DLPFC, the amygdala, the insula, and the fusiform gyrus. It should be noted that there was considerable variability across DCM studies with regards to the number of specified models, the included regions, their intrinsic connections, their driving inputs, and their task-factor modulating connections (see the Results column of Table 1 under the Effective Connectivity section for details of specified models).

Three studies found that frontal regions exerted a modulatory effect on subcortical and occipital regions (da Silva et al., 2010; Sladky et al., 2015; Torrisi et al., 2013). For example, Da Silva et al. (2010) found that when passively viewing sad faces (relative to neutral faces), the DLPFC exerted a modulatory effect on the fusiform gyrus and the anterior cingulate cortex. Similarly to these studies, but employing an emotion regulation task, Morawetz et al. (2015) found that the inferior frontal gyrus (IFG) exerted an inhibitory effect on DLPFC activity. Conversely, Dima et al. (2015) found that angry and fearful faces strengthened the forward connection from the inferior occipital gyrus (IOG) to the ventral prefrontal cortex (VPFC). Similarly, Dima et al. (2011) also found that when recognizing angry faces (relative to neutral faces), the forward connection from the IOG to the VPFC was strengthened. This study built upon the finding from an earlier DCM study in which viewing happy and fearful faces increased forward connectivity from the Inferior occipital gyrus to the fusiform gyrus and the amygdala (Fairhall and Ishai, 2006). This finding was explained as representing the hierarchical structure of the ‘core’ and ‘extended’ face perception networks. The ‘core’ network is composed primarily of visual regions, which has a feed-forward influence on the ‘extended’ face perception network responsible for emotional and social aspects, and which includes limbic and prefrontal areas.

Although primarily interested in memory encoding of emotional stimuli, and examining a model solely composed of the amygdala and hippocampus, Fastenrath et al. (2014) conducted a well-designed, high-powered study whose findings are relevant to understanding emotion processing. While encoding positive and aversive images, participants exhibited an increase in connectivity strength projecting from the amygdala to the hippocampus, regardless of the emotional valence. This pattern was not observed while participants encoded neutral images. Furl et al. (2013) also found the amygdala crucial to processing the salience of emotional stimuli. While participants viewed static and dynamic happy, fearful, and disgusted faces, the amygdala exerted a top-down effect on visual areas such as the occipital face area, the middle temporal visual area, and the fusiform gyrus. These visual areas initially received external input from the presented faces, which the amygdala then modulated. Therefore, the amygdala appeared to optimize visual processing of each stimulus depending on its emotional valence and mode of presentation (static vs dynamic).

Mazzola et al. (2016) modeled connections between the insula and the superior temporal gyrus. The insula is less extensively investigated relative to the amygdala, but which nonetheless is likely involved in processing emotional salience (Cauda et al., 2012; Phillips et al., 2003), particularly disgust and anger (Fusar-Poli et al., 2009). This study focused on the posterior insula, which is hypothesized to process interoceptive, emotional and environmental information, which the anterior insula then evaluates for potential salience (Cauda et al., 2012). Participants watched videos of actors grasping an object with an angry or joyful expression. Angry expressions, when compared to neutral ones, resulted in an increased forward connection from the insula to the right superior temporal gyrus, but suppressed the projection to the left superior temporal gyrus.

Finally, five studies found that affect modulated connections between frontal and subcortical regions bidirectionally (Breakspear et al., 2015; Goulden et al., 2012; Tak et al., 2021; Vai et al., 2015; Willinger et al., 2019). One of these studies found that the directionality of effective connectivity differed when contrasting responses to stimuli with positive and negative valences. Willinger et al. (2019) modeled a network composed of the amygdala, MPFC, LPFC, and FG to examine responses during a face-matching task involving dynamic faces. The authors found that the top-down connection from the MPFC to the amygdala was modulated by both positive and negative faces, while the bottom-up connection from the amygdala to the MPFC was modulated by negative and neutral faces only. Furthermore, the connection from the LPFC to the MPFC was modulated by positive and negative faces.

## 4 Discussion

To date, the present systematic review represents the first to consider fMRI connectivity findings in negative emotion processing and emotion regulation in the general population. The findings extend existing reviews of functional activation by showing connectivity between multiple regions predicted to co-activate during emotion processing. Contrary to claims made by existing models, there was mixed support for previously proposed top-down hierarchical models of emotion regulation, investigated by more recent studies able to make inferences about the directionality of connectivity.

### 4.1 Emerging findings

Despite variation in study design, methodology, and quality, some generalities were observed. The majority of studies chose the amygdala as a region of interest for their connectivity analyze. In a meta-analysis, the amygdala was found to respond to salience, regardless of valence (Sergerie et al., 2008). The findings in the present review support this view. Nonetheless, it should be noted that there is evidence of a subcortical ‘shortcut’ between amygdala and pulvinar facilitating fear recognition (McFadyen et al., 2019), thus it remains an important region for negative emotion and threat processing.

There was support for existing models of emotion processing, involving the amygdala, the insula, the anterior cingulate cortex, the inferior frontal gyrus, the orbitofrontal cortex, and the dorsolateral prefrontal cortex. Interestingly, there was considerable overlap in the regions reported and their purported connectivity across emotional valence processing and emotion regulation studies, as visually summarized in Figs. 2 and 3. There was not always consistency as to whether connectivity increased or decreased between these regions during the tasks in each study. This in part reflects variation across studies with regards to task demands. Nevertheless, this is a novel finding that arguably challenges the assumption of increased connectivity during emotion processing put forward by models derived from activation studies (Buhle et al., 2014; Ochsner et al., 2012; Phillips et al., 2003).

In line with claims made by previous models of emotion regulation (Ochsner et al., 2012; Phillips et al., 2003), the findings generally supported the assumption that effortful up- or down-regulation of emotion involves changes in connectivity particularly between the amygdala, the IFG, and the DLPFC. However, there was one notable exception, below.

### 4.2 Implications of effective connectivity findings for top-down accounts of emotion processing

The findings from studies employing measures of effective connectivity allow for claims to be made regarding the directionality of connectivity changes during emotion processing. Figs. 2 and 3 include arrows representing directional influences within the network found across these studies. Note that the influence of the IOG on the VLPFC during categorization of negative faces found by Dima et al. (2011) was omitted, as the VLPFC was not commonly included in the studies reviewed. Viewing these directional relationships in Fig. 2 reveals a combination of top-down, bottom-up, and bidirectional influences on the connections between cortical and subcortical regions. Hence, there was greater support for a dynamic, rather than hierarchical understanding of the driving influences between regions in the network. For example, (Morawetz et al., 2015) found that the IFG inhibited the DLPFC during an emotion regulation task involving extreme sports clips. This would imply more complex feedback mechanisms between frontal areas separate to the connections with the amygdala that prominent models consider key to emotion regulation (Ochsner et al., 2012; Phillips et al., 2003). The authors posited that the DLPFC may hold different reappraisal goals in working memory (*e.g*. up or down-regulation), while the IFG selects the appropriate reappraisal, inhibiting the DLPFC once it has done so. Breakspear et al. (2015) used non-linear DCM analysis to show that the ACC and DLPFC gate each other's influences on the IFG with regards to fear and cognitive control (*i.e*. go vs no-go), with the ACC and DLPFC influenced in turn by connections from the fusiform gyrus.

Altogether, this would support the theory that causal relationships between regions shift as a function of task demands (Pessoa, 2017). These context-dependent changes in effective connectivity may also relate to the emotional valence of the stimuli being presented. During a face-matching task, Willinger et al. (2019) found both top-down and bottom-up modulatory influences on the connections between prefrontal areas and the amygdala, depending on whether participants viewed positive, neutral, or negative emotional faces. Altogether, these findings suggest a model of emotion processing with dynamic, context-dependent modulatory relationships between primarily limbic and prefrontal areas, rather than a hierarchical, top-down model as previously proposed. Predictive processing models hypothesise that these dynamic relationships stem from continual feedback signals between top-down and bottom-up regions (Seth and Critchley, 2013; Barrett and Simmons, 2015). Bottom-up interoceptive prediction errors (predictions about the physiological condition of the body) are updated by cascading top-down interoceptive predictions, and it is the integrated result of this feedback which gives rise to subjective emotion (Seth et al., 2012). This might help explain the observed patterns in connectivity and driving influence from both top-down and bottom-up regions in the present review.

### 4.3 Quality of included studies

We derived a tool evaluating the quality of neuroimaging studies included in this review. The majority of studies presented clear methods with sample sizes reaching the proposed minimum for achieving adequate statistical power (Friston, 2012). There was variation across studies in terms of the emotion processing task selected, but also the model structures and the number of possible modulatory relationships tested. There was some consistency in the regions included into model spaces, with the amygdala, FG, IFG, and IOG frequently involved. The common practice of including the amygdala as a region of interest underscores its central theoretical importance for facial emotion processing, which was supported by our review. However, considerable variability existed with regards to other included regions and their proposed modulatory effects, which may ultimately lead to both key regions and connections being excluded. Furthermore, there was a general lack of anatomical specificity reported in the included studies, despite increasing evidence that many regions, *e.g*. the insula (Allen, 2020; Perry et al., 2019; Singer et al., 2009), can be subdivided into areas specialized for distinct emotional processes. Only six studies labeled specific regions within the ACC in their connectivity findings (Banks et al., 2007; Das et al., 2005; Hakamata et al., 2016; Morawetz et al., 2017; Raz et al., 2016; Williams et al., 2006), despite evidence of subdivisions for cognitive and emotional function (*e.g*. dorsal vs. rostral ACC; Mohanty et al., 2007). Many key regions such as the DLPFC and insula were associated with widely varying coordinates across the included studies, as may be seen in Figs. 4 and 5.

### 4.4 Recommendations and future directions

Given the limitations described earlier, some methodological recommendations can be made. As noted, there was heterogeneity across studies in terms of task designs, imaging methods, analyze, regions of interest, and processes of interest. This may in part reflect funding and time constraints driving researchers towards novelty over replicability (Carp, 2012). Nonetheless, replication studies are key to establishing reliable experimental effects. It is also important that both replication and novel studies recruit large sample sizes, since small sample sizes and associated low statistical power are prevalent in the current literature (Fusar-Poli et al., 2009). Appropriate statistical thresholding is also important given the number of comparisons typical in fMRI studies, although the majority of included studies in the present review employed appropriate thresholding. Other recommendations for future studies that apply to all experimental research include pre-registration and registered reports (Mehler, 2019).

Characterising ‘normal’ or ‘healthy’ populations may aid in the development of normative functional maps of emotion processing. Such maps may help contextualise alterations observed in individuals with clinical disorders such as depression or psychosis (Stuhrmann et al., 2011; Underwood et al., 2015), by helping distinguish neurobiological dysfunction from methodological inconsistencies (Fusar-Poli et al., 2009). Of relevance is the observation that detailed participant demographics were infrequently reported amongst the included studies. The potential confounding effects of such factors (*e.g*. age; Fischer et al., 2005) on activation and connectivity are not fully understood, and future studies may wish to stratify their sample by different demographic variables during analysis.

Only a small number of studies examined connectivity using methods that capture the direction of influence. Furthermore, the variability with regards to model space across these studies limits their comparability. These analyze are nonetheless important because they help directly examine previously assumed directional influences between regions featured in existing models of emotion processing. The emerging findings in the present review suggest two main points to consider for future research. The first is that, rather than cortical regions ‘controlling’ subcortical ones, the directional relationships between these regions may be bidirectional. The second is that the directionality of these relationships may be dynamic, and context-dependent. Further exploration of changes in effective connectivity due to context, such as stimulus valence, will be crucial for further elucidating the functional organization of emotion processing networks. Authors may also wish to make use of advances in the applications of DCM, including non-linear DCM (Stephan et al., 2008), and Parametric Empirical Bayes for DCM (Friston et al., 2002).

Of note, while the majority of studies used the same software package for running their analyze (Statistical Parametric Mapping), there was variation in the version of SPM used, and some studies employed other analysis software such as FSL. Although difficult to estimate in the present review, it is possible for different software packages to produce discrepant findings for the same analyze (Bowring et al., 2019). Future studies may wish to repeat their analysis pipeline in multiple software packages and report any divergence in results.

### 4.5 Conclusion

The present review provided empirical validation for previously proposed models of emotion processing and the assumptions made about connectivity within the network. This review also summarized evidence regarding the driving influence of different regions involved in negative emotion processing and emotion regulation. This evidence challenged the assumption that it is mainly prefrontal areas that exert directional effects on other regions. Ultimately, the findings in the present review indicated that the modulatory relationships between regions involved in emotion processing may be more nuanced than previously claimed.

## Appendix Supplementary materials

Image, application 1
