# Supplementary material for: Networks underpinning emotion: A systematic review and synthesis of functional and effective connectivity
Source: Neuroimage. 2021 Nov;243:118486. doi: 10.1016/j.neuroimage.2021.118486 (PMC8905299; doi:10.1016/j.neuroimage.2021.118486)
Supplement: Supplementary file 1 [file mmc1.docx]

# Supplementary materials

### Description of quality rating tool and list of items

The first 5 items of the tool are applicable to experimental studies regardless of the use of neuroimaging techniques. These items were taken directly from the NIH Quality Assessment Tool for Observational Cohort and Cross-Sectional Studies (Health, 2014). The remaining five items were developed using recently published, highly cited papers on fMRI methodology authored by leaders in the field (Brooks et al., 2017; Eklund et al., 2016; Friston, 2012; Poldrack et al., 2008). Apart from items 7, 8 and 9, all items require a yes/no response. Items 7 and 8 require a yes/no response, as well as a further explanation if the response is ‘no’. Item 9 requires a response of either ‘a priori’ or ‘data-driven’. No items require a further graded assessment of quality, such as good/fair/poor. This decision was made as although there are recently published papers advocating ideal fMRI study design and analysis (David et al., 2013; Poldrack et al., 2008), there are not currently widely agreed categories of quality within the neuroimaging literature.

Items taken from NIH Quality Assessment Tool for Observational Cohort and Cross-Sectional Studies:

1. Was the research question or objective in this paper clearly stated?

2. Was the study population clearly specified and defined?

3. Was a sample size justification, power description, or variance and effect estimates provided?

4. Were the exposure measures (independent variables) clearly defined, valid, reliable, and implemented consistently

5. Were the outcome measures (dependent variables) clearly defined, valid, reliable, and implemented consistently across all study participants?

Neuroimaging-specific items:

6. Was the sample size above 16?

7. Was the activation analysis corrected for multiple comparisons (e.g. using Family Wise Error or False Discovery Rate)? If not, how was this justified?

8. Was the connectivity analysis corrected for multiple comparisons (e.g. using FWE or FDR)? If not, how was this justified?

9. Were regions of interest (ROIs) defined a priori, or data-driven?

10. Were stimuli (e.g. emotional faces) checked behaviourally for valence either during or after the scan?

### Supplementary table of quality ratings

| Quality item | 1 (Y/N) | 2  (Y/N) | 3  (Y/N) | 4  (Y/N) | 5  (Y/N) | 6  (Y/N) | 7 | 8 | 9 | 10  (Y/N) |
| --- | --- | --- | --- | --- | --- | --- | --- | --- | --- | --- |
| Study |  |  |  |  |  |  |  |  |  |  |
| Banks et al. (2007) | Y | Y | N | Y | Y | N | N, no justification | N, no justification | A priori | Y |
| Blasi et al. (2009) | Y | Y | N | Y | Y | Y | Y | Y | A priori, data defined | Y |
| Breakspear et al. (2015) | Y | Y | N | Y | Y | Y | Y | N/A (DCM) | Data driven | N |
| da Silva et al. (2010) | Y | N | N | Y | Y | Y | Y | N/A (DCM) | A priori | N |
| Das et al. (2005) | Y | Y | N | Y | Y | Y | N, justified by novelty | N, justified by novelty | A priori | Y |
| De Marco et al. (2006) | Y | Y | N | Y | Y | N | N, no justification | N, no justification | A priori | N |
| Denny et al. (2014) | Y | Y | N | N | Y | Y | Y | Y | A priori, data defined | Y |
| Dima et al. (2011) | Y | Y | N | Y | Y | Y | Y | N/A (DCM) | A priori | Y |
| Dima et al. (2015) | Y | Y | N | Y | Y | Y | Y | N/A (DCM) | A priori | Y |
| Fairhall & Ishai (2006) | Y | N | N | N | Y | N | N, no justification | N/A (DCM) | A priori, data defined | N |
| Fastenrath et al. (2014) | Y | Y | N | Y | Y | Y | Y | N/A (DCM) | Data driven | Y |
| Furl et al. (2013) | Y | N | N | Y | Y | N | Y | N/A (DCM) | Mixed | Y |
| Goulden et al. (2012) | Y | Y | Y | Y | Y | Y | N, no justification | N/A (DCM) | A priori | N |
| Hakamata et al (2016) | Y | Y | N | Y | Y | Y | Y | N, justified by novelty | A priori | N |
| Hyrbouski et al. (2016) | Y | Y | N | Y | Y | Y | Y | Y | A priori | Y |
| Jabbi & Keysers (2008) | Y | N | N | N | Y | Y | N, no justification | N, no justification | A priori, data defined | N |
| Mazzola et al (2016) | Y | Y | N | N | Y | Y | Y | Y | A priori | N |
| Miyahara et al (2013) | Y | Y | N | N | Y | Y | Y | Y | A priori | Y |
| Morawetz et al (2015) | Y | Y | N | Y | Y | Y | Y | N/A (DCM) | peak activation | Y |
| Morawetz et al (2017) | Y | Y | N | Y | Y | Y | Y | Y | A priori | N |
| Park et al. (2016) | Y | Y | N | Y | Y | Y | Y | N/A (graph theoretical analysis) | A priori | N |
| Payer et al. (2012) | Y | Y | Y | Y | Y | N | N, no justification | N, no justification | A priori | N |
| Raz et al. (2016) | Y | Y | N | Y | Y | Y | Y | Y | A priori | Y |
| Satterthwaite et al. (2011) | Y | Y | N | Y | Y | Y | Y | Y | A priori | Y |
| Schienle & Scharmuller (2013) | Y | Y | N | Y | Y | Y | Y | Y | Data driven | Y |
| Sladky et al. (2015) | Y | Y | N | Y | Y | N | N, no justification | N/A (DCM) | A priori | Y |
| Sripada et al. (2014) | Y | Y | N | Y | Y | Y | N, justified by connectomic PPI | N, justified by connectomic PPI | Data driven | Y |
| Tak et al. (2021) | Y | Y | N | Y | Y | Y | N, no justification | N/A (DCM) | A priori | N |
| Torrisi et al. (2013) | Y | Y | N | Y | Y | Y | N, no justification | N/A (DCM) | A priori | Y |
| Tschacher et al. (2010) | Y | N | N | N | Y | Y | N, novelty given as reason | N, novelty given as reason | A priori | Y |
| Vai et al. (2015) | Y | Y | N | Y | Y | Y | N, no justification | N/A (DCM) | A priori | N |
| Williams et al. (2006) | Y | Y | N | Y | Y | N | N, justified by random effects model | N, justified by random effects model | A priori | Y |
| Willinger et al. (2019) | Y | Y | N | Y | Y | Y | Y | N/A (DCM) | A priori | Y |
|  |  |  |  |  |  |  |  |  |  |  |
